# Attractiveness consumption, personality traits and sustainability: Construction and empirical application of evaluation indicators for attractive attributes of China-chic T-shirt products

**DOI:** 10.3389/fpsyg.2022.1101978

**Published:** 2022-12-30

**Authors:** Hongwen Liu, Chunli Guo, Beibei Zhang

**Affiliations:** ^1^School of Arts, Soochow University, Suzhou, China; ^2^School of Fashion Design, Jiangxi Institute of Fashion Technology, Nanchang, China

**Keywords:** China-chic T-shirt products, attractive attribute evaluation, index system, decision-making personalities, sustainability

## Abstract

**Background:**

The rapid development of China’s cultural and creative industries has given cultural fashion products, represented by China-chic (国潮) T-shirts, vitality. In recent years, with the arrival of the era of design-led emotional sensory, products with attractive and qualia characteristics have become an important tool to attract consumers and create a competitive brand.

**Objective:**

Therefore, this study aimed to build an evaluation index of the attractive attributes of China-chic T-shirt products from the perspective of attractive consumption to serve as an important basis for improving product development and thus promoting the sustainable development of China-chic T-shirt products.

**Methods:**

Firstly, a three-level hierarchical model was established for the sustainable development of China-chic T-shirt products based on literature research. Secondly, through the evaluation grid method and factor analysis method, we extracted the attractive elements and factors associated with China-chic T-shirt products and established an objective evaluation index for the attractive attributes of China-chic T-shirt products. Twenty-five participants with different decision-making personalities tested three products with expert validity. The weights of the evaluation indexes of the attractive attributes of China-chic T-shirt products were evaluated using the fuzzy analytic hierarchy process. Finally, a two-factor mixed design ANOVA was conducted to check the practical value of the evaluation index of the attractive attributes of China-chic T-shirt products.

**Results:**

The results show that the evaluation indexes of the attractive attributes of China-chic T-shirt products include 7 attractive factors, including the brand image feature, perceptual association feature, beauty feature, delicacy feature, creativity feature, engineering feature, and green feature, as well as 32 attractive elements. Through an empirical application analysis, it was found that the index has an excellent ability to differentiate between the attractive characteristics of different styles of products and test participants with different decision-making personalities.

## 1. Introduction

While consumers fully appreciate the convenience brought by the functional attributes of a product, they are more concerned than ever about their own perceptual needs in terms of consumption, and their motivation to ultimately purchase a product also depends on the degree to which the product satisfies their spiritual preferences (Alaniz and Biazzo, 2019). In recent years, China-chic clothing has attracted significant attention due to its distinctive fashion style and Chinese cultural characteristics. As the core item in China-chic clothing, the T-shirt has a distinctive and unique style. This comes from the designer, who has mastered the product’s personality by integrating cultural elements into the design and letting the product’s personality reveal the cultural temperament and connotations, thereby satisfying the consumers’ mental and emotional needs. This is the biggest difference between these products and ordinary T-shirt products and is an important reason for consumers to choose the China-chic T-shirt products (Zhang et al., 2022). As He et al. (1996) argued, compared with general products, cultural and creative goods give consumers a pleasant feeling and stimulate their psychological emotions through spiritual and cultural factors, beauty forms, intrinsic meanings, cultural symbols, and other attractive and qualia factors, which in turn trigger purchase behaviors. Additionally, as Yan et al. (2014) suggested, cultural and creative products elicit psychological responses that differentiate cultural and creative products from general products. Therefore, based on the viewpoints of the above scholars, in the design of China-chic T-shirt products, in addition to meeting the functional demands, designers should pay attention to the unique emotional experience that the product brings to consumers at the level of attractiveness and qualia.

Product evaluation is an important link in modern product design activities, and it is also an effective means to avoid risks and improve product pertinence (Quan et al., 2019). Moreover, in recent years, with the rapid development of the China-chic T-shirt industry, problems such as misalignment between design and demand and product homogenization have occurred from time to time. One of the reasons for this is that there is no unified and objective product evaluation index available in the current industry. The determination of the final design products of most China-chic T-shirt companies is basically a comprehensive assessment of the products by departmental evaluators through their personal perspectives. The assessment process is often somewhat subjective and uncertain (Shi and Bai, 2022). Therefore, starting from attractive consumption, we constructed an objective evaluation index for the attractive attributes of China-chic T-shirt products to explore the common language between design supply and demand. Through continuously optimizing the design quality of the consumer object, the design should be able to satisfy the spiritual experience and esthetic taste needs of consumers, so that sustainable development is achieved through the mutual promotion of the two. However, current theoretical research on the design of China-chic T-shirt products is still mostly based on subjective research from the designer’s perspective, and there has been less theoretical research focusing on actual consumer needs. For example, Ma and Wang (2020) explored a new way of designing cultural and creative T-shirts by using Terracotta Warriors as elements from the perspective of cultural communication in order to better realize the role of these products as a medium for the communication and promotion of the Terracotta Warriors culture. On the other hand, Zhang and Zhang (2014) divided the creation process of traditional hand-painted T-shirt patterns into two stages, design and drawing, and proposed an operation method of combining traditional hand-painting techniques with a knitted T-shirt pattern design. Huang (2013) explored the effect of including cultural symbols of cultural tourism attractions on promotional T-shirt products based on a design crossover perspective and provided specific design examples. Among the existing research studies, there are a few theoretical studies based on consumer demand. However, the focus of these studies is also mainly on the esthetic evaluation of products by consumers using characteristics such as color, pattern. and structure (Wang and Liu, 2014; Deng and Wang, 2018), and such research lacks the in-depth excavation of consumers’ inner preferences and the visual presentation of products’ attractive factors.

Given this, this study firstly analyzed the attractive characteristics of China-chic T-shirt products by adopting the evaluation grid method used by Miryoku Engineering. This method is a systematic method for identifying the attractive elements of various products. In practical applications, this method can be used to fully consider user needs and enhance user participation in product design, effectively avoiding subjective decision-making by enterprises and designers on design solutions. On this basis, the exploratory factor analysis method was applied to identify the common attractive elements of China-chic T-shirt products. The exploratory factor analysis method represents the original data structure with fewer dimensions while preserving most of the information provided by the original data structure. Therefore, it is suitable for sorting out the inner structure of attractive elements to achieve the purpose of streamlining the scale and then completing the construction of an evaluation index of the attractive attributes of China-chic T-shirt products. Finally, through a practical case study and a combination of fuzzy analytic hierarchy process and two-factor mixed-design ANOVA, a sensitivity difference analysis was conducted to verify the practical value of the evaluation index of the attractive attributes of China-chic T-shirt products.

## 2. Literature review

### 2.1. Sustainability of China-chic T-shirt products in terms of attractive consumption

**Figure 1:**
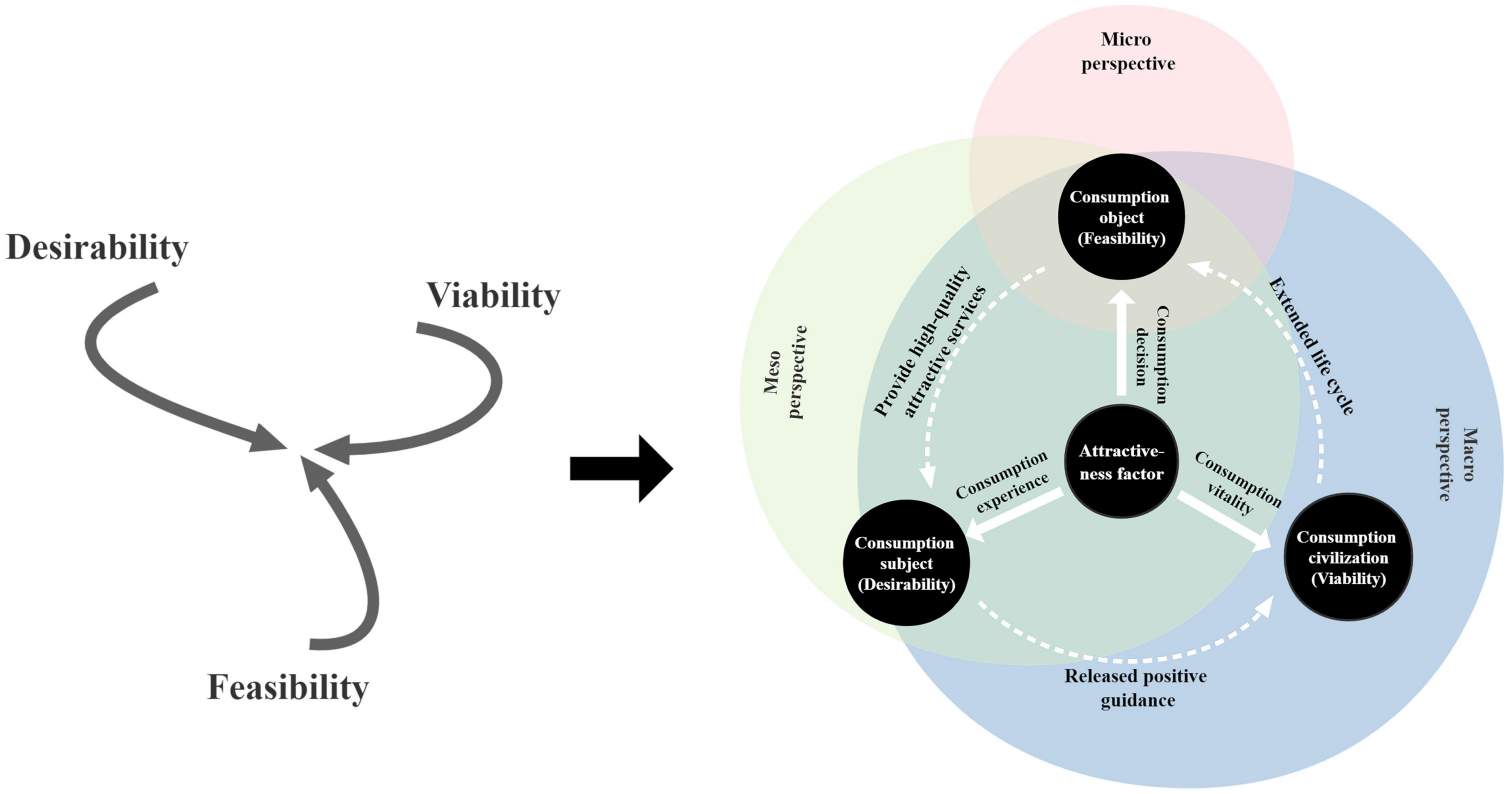
The three-level hierarchical model for the sustainable development of China-chic T-shirt products. Drawn by author.

In their article “Change by Design,” Brown and Barry (2011) proposed three limitations that affect the sustainable development of product design and overlap with each other, namely feasibility (the feasibility of product functions in application), desirability (valuable to users and able to capture the hearts of consumers), and viability (products have the potential to be part of a company’s sustainable business model). They point out that a competent designer will solve these three limitations one by one, and a design thinker will allow these three limitations to reach a state of harmony and balance. At the same time, Chen and Yang (2022) suggested that if product design is only focused on the economic value of a product, the consumer experience will be neglected, resulting in only short-term benefits in economic terms. The idea of sustainable consumption lies in the pursuit of a balance between the object of consumption, the subject of consumption, and the civilization of consumption. Under the guidance of this idea, the design of products should not only focus on the current stable development but also consider the endless consumption benefits generated by continuous product innovation, combining current and long-term interests. Based on this, many scholars have actively explored how to promote the generation of products with sustainable consumption benefits. Among them, in his famous the theory of attractive quality, Japanese quality management master Noriaki et al. (1984) stated that attractive quality is a necessary condition for gaining customer loyalty. He pointed out that customers are attracted to products essentially because of the high quality of their attractive elements, as this brings them a constant sense of surprise and pleasure. It keeps customers hooked on the product and generates continued purchasing behaviors. Given this, this study used the research findings of Tim Brown, Barry Katz, Chen Xiang, and Noriaki Kano to introduce the idea of a three-level hierarchy system incorporating micro, meso, and macro sustainable products proposed by Yao (2020). From the perspective of product attractive consumption, the attractiveness factor was taken as the core driving force to promote the balance between the consumption object (feasibility), consumption subject (desirability), and consumption civilization (viability), and a sustainability hierarchy model for the China-chic T-shirt product was established (as shown in Figure 1).

From the point of view of attractive consumption, the sustainable development of China-chic T-shirt products can be analyzed from four aspects: the consumption object under the micro perspective, the consumption subject under the meso perspective, the consumption civilization under the macro perspective, and the core driving force “attractiveness factor” throughout the three levels. From a micro perspective, China-chic T-shirt products, as the consumer objects, are the material carrier and basic link in this transmission process. In the design process, designers need to focus on extracting and expressing the attractive characteristics of the products to enhance their storytelling attributes, attractiveness, and inner meaning. The micro perspective focuses on addressing the feasibility limitations. From the meso perspective, as consumer objects, the expression of the attractive attributes of China-chic T-shirt products should be analyzed and considered in a more comprehensive way by incorporating the dimension of interaction with consumers. For example, differentiated expressions should be used for the characteristics of different consumers (such as different decision-making personalities, age, etc.) in order to promote the correct interpretation of the products’ attractiveness symbols through the use behaviors of consumers and consolidate the connection between consumer objects and subjects. The meso perspective model further pursues the balance between the two limitations of feasibility and desirability. Consumption civilization, from the macro perspective, is composed of consumption objects, consumption subjects, and attractive consumption. In an excellent sustainable cycle of attractive consumption, the consumed China-chic T-shirt products must be able to produce a positive guiding effect on the consumption subject and release this effect into the overall circulation system, thus stimulating the generation of new consumption behaviors by the consumption subjects. Under the influence of a sustainable consumption civilization, consumers can generate multiple consumption actions, allowing the life cycle of a consumer object to be extended. The macro perspective model can truly achieve a harmonious balance between the three limitations of feasibility, desirability, and viability.

Therefore, regarding China-chic T-shirt products, the company should not only strive to achieve a high level of business performance and provide consumers with a good consumption experience in terms of design, they should also ensure that the two elements promote each other as well as the sustainability of the development process of the China-chic T-shirt industry. This study focused on the core project, the extraction and evaluation of the attractive factors associated with China-chic T-shirt products, to lay the foundation for subsequent integrated research.

### 2.2. Miryoku engineering and evaluation grid method

#### 2.2.1. Miryoku engineering

Many questions about attractiveness arise when creating new products, and determining how to extract the attractiveness of a product is an element that many designers strive to find. However, “attractiveness” is a vague concept that is difficult to evaluate specifically. Therefore, in 1991, the Japanese scholar Masato UJIGAWA gathered a number of scholars and launched a study on Miryoku engineering products with the aim of using “Technology and knowledge to create attractive products and spaces” (Asahino, 2001; Wei and Ma, 2020). Miryoku Engineering is a design concept based on consumer preferences that enables a communication bridge to be established between designers and consumers. The main research method used by Miryoku Engineering is the evaluation grid method.

#### 2.2.2. Evaluation grid method

**Figure 2:**
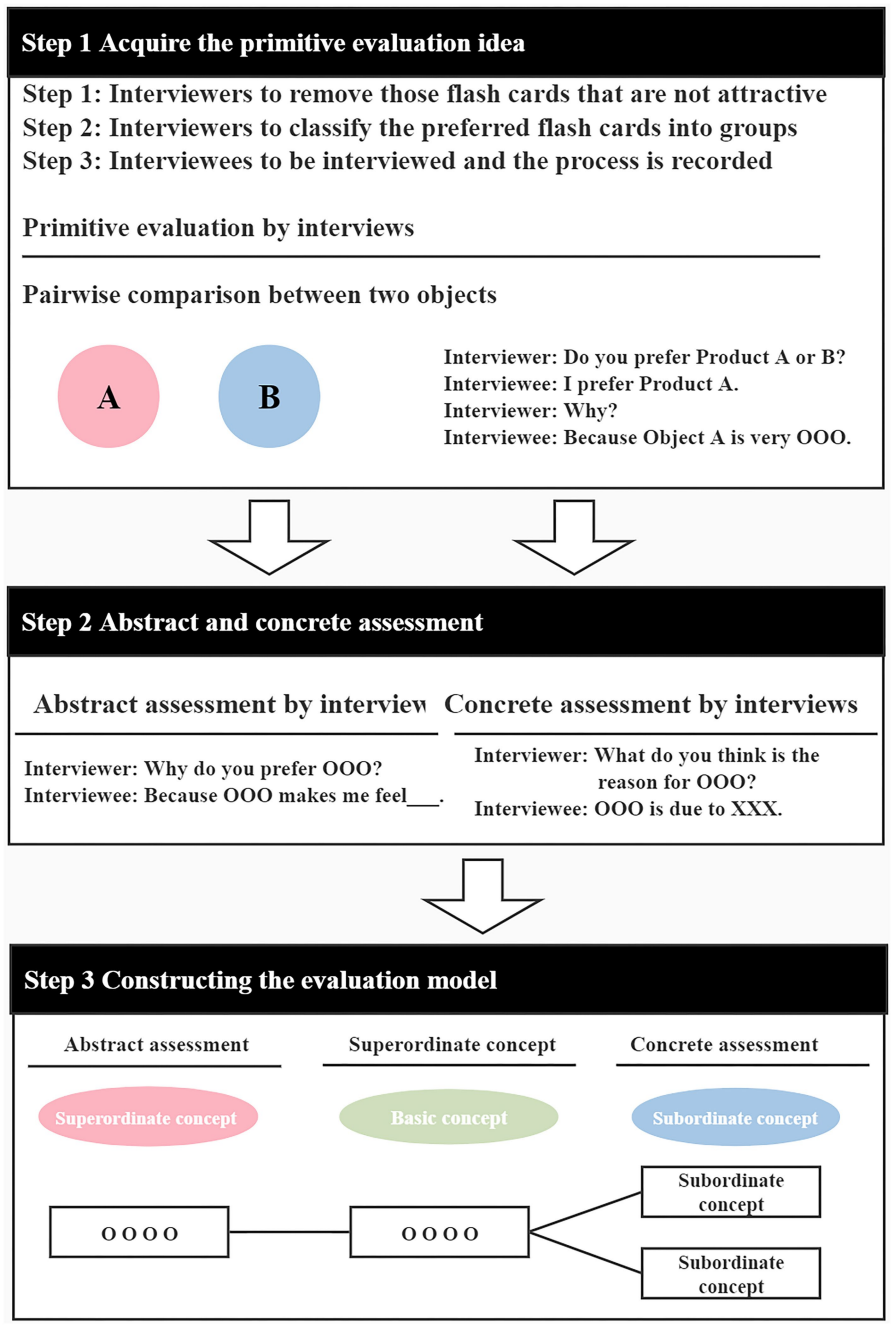
The implementation procedure used in the evaluation grid method. Redrawn from Ko et al. (2018) (CC-BY) (http://creativecommons.org/licenses/by/4.0/).

The Japanese scholar Junichiro Sanai developed the evaluation grid method by studying the repertory grid method proposed by KELLY and improving it. This method can help the researcher to gain insight into a subject’s psychological perception of something (Kelly, 1955). The evaluation grid method is based on the comparison of a pair of objects, A and B, through personal interviews, followed by a compilation of the individual attractive characteristics of the target objects after clearly discussing the similarities or differences between the objects (Ma et al., 2011). The implementation of this method is mainly divided into two steps: first, in the evaluation of the target object, the respondent needs to answer questions about their likes or dislikes for the object. The second step is to clarify the meaning or condition of the respondents’ answers through the use of additional questions to specifically analyze the factors of a product that attract consumers and to organize the related structural network (Sanui, 2000; As shown in Figure 2). The attractiveness factors obtained by the evaluation grid method include three items, namely, the median item (original assessments), the upper item (abstract reasons), and the lower item (concrete reasons).

The evaluation grid method has been successfully used in product design and development. Especially in recent years, the advent of the design-led emotional sensory era has led to more widespread use of the evaluation grid method in various product design research fields. For example, Chen et al. (2012), Shen (2013), Park et al. (2016), Chang and Chen (2017), Ko et al. (2018), Takada et al. (2019), and Li and Wen (2021) used the evaluation grid method to extract the attractiveness factors associated with SNS games, mobile hospital applications, action figures, office chairs, automobiles, and furniture products, respectively, in order to accurately identify user needs and preferences as a reference for design. In the field of apparel product research, Ma et al. (2012) and Tu and Chang (2017) used the evaluation grid method to study the attractiveness factors associated with wedding dress products; Lin et al. (2011) applied the method to extract the attractiveness factors associated with popular clothing; Ruan and Lin (2016) explored the attractive attributes of uniform design based on the evaluation grid method, and Chen and Ming (2019) used the evaluation grid method to study the most attractive characteristics of cheongsam products. In addition, in research on accessories, Lu et al. (2022) combined the Miryoku engineering and importance-performance analysis methods to conduct design research on the Chinese bride’s phoenix crown. Chia-Hui et al. (2020) applied the evaluation grid method to the appearance evaluation of leather crafts.

The above literature review shows that the Miryoku engineering evaluation grid method is being applied to increasingly rich application scenarios in the field of clothing, but the following problems still exist: Firstly, from the viewpoint of the research object, since different types of apparel products have different constituent elements in terms of their attractiveness attributes, it is of practical significance to conduct research on specific products. However, current research on the attractiveness attributes of China-chic T-shirt products is still lacking, so corresponding research needs to be supplemented. Secondly, from the perspective of research, the above-mentioned scholars’ research mainly focuses on the extraction of product attractiveness factors and their design practice guidance while lacking in-depth consideration of consumers’ decision-making personalities and verification of the practical value of product attractiveness factors. Thirdly, in terms of research methods, the methodology used in the above study is dominated by the evaluation grid method combined with the quantification theory type I method, leading to the problem of having a single method of application and leading to a lack of credibility in the research conclusions. Based on this, to promote the sustainable development and consumption of China-chic T-shirt products, this study comprehensively applied a variety of qualitative and quantitative research methods to construct an evaluation index for the attractive attributes of China-chic T-shirt products. In the application verification part, in order to test the practical value of the evaluation indexes, we screened consumer groups with different decision-making personalities for comparison to enhance the practical application validity of the research results.

## 3. Construction of an evaluation index of the attractive attributes associated with China-chic T-shirt products

**Figure 3:**
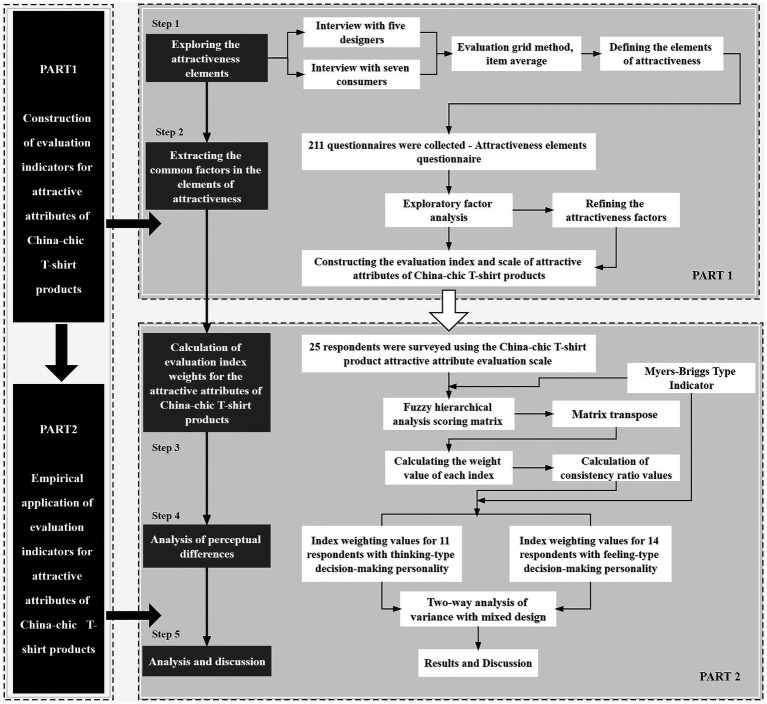
The research structure and process used in this study. Drawn by author.

The purpose of this study was to construct an evaluation index of the attractive attributes of China-chic T-shirt products and to assess its practical application value. In the evaluation and validation part of the experiment, three China-chic T-shirt products with expert validation were used as the experimental samples, and two consumer groups with different decision-making personalities, thinking types, and feeling types were selected to analyze the perceptual differences in the attractive attribute evaluation index. The specific research steps were divided into three stages: In the first stage, based on the determination of the experimental product and the interviewees, in-depth interviews were conducted using the evaluation grid method from Miryoku engineering theory to extract the attractiveness elements associated with the China-chic T-shirt product and establish an evaluation construction diagram. In the second stage, a larger-scale questionnaire based on the concrete reasons summarized in the first stage of the evaluation grid method was conducted. Subsequently, the factor analysis method was used to extract the common factors in the attractiveness elements to complete the construction of the evaluation index of the attractive attributes associated with China-chic T-shirt products and to develop the scale of the evaluation index of the attractive attributes of China-chic T-shirt products. In the third stage, questionnaire interviews were conducted with 25 people based on this index scale. At the same time, the Myers–Briggs Type Indicator (MBTI) was used to identify two types of consumers classified by their thinking and feeling tendencies in terms of decision-making patterns. Subsequently, the fuzzy analytic hierarchy process was applied to calculate the weights of the evaluation indexes of the attractive attributes of these two types of consumer groups in relation to China-chic T-shirt products with different design styles. Finally, a two-factor mixed design ANOVA was applied to perform a perception difference analysis. The research structure and process of this study are shown in Figure 3 below.

### 3.1. Exploration of the attractiveness elements of China-chic T-shirt products

In order to clarify consumers’ preferences and needs regarding China-chic T-shirt products and to identify the key attractiveness elements of these products, this paper explored China-chic T-shirt products using the evaluation grid method and analyzed consumers’ specific evaluations of the desired products.

#### 3.1.1. Sample of experimental products

This paper selects some of the China-chic T-shirt products being sold in the current market as tested products. The screening process included the following three processes:

Selection of the experimental brands: with reference to the comprehensive indicators such as brand awareness, brand style, the status of the brand’s online published performance and experts’ opinions in the field, this study classified the selected China-chic T-shirt brands. The first category was innovative brands, and this included 9 design-oriented brands: Mukzin, INXX, ICY, Bosie Agender, MAGMODE, HUSENJI, HUAMUSHEN, BYTEHARE, and Panda Store. Most of these brands have successfully obtained Series A or Series B financing, for example, Mukzin received tens of millions of dollars in Series A financing led by CapitalNuts in 2018, which also indicates its good development momentum. The second category was traditional reinvented brands: China Lining, Anta, Heilan Home, Peacebird, and Day Broadcast. In recent years, these traditional domestic well-known brands have begun to emphasize their sense of design to promote the national trend as a way to attract the younger generation. The third category was the classic brand type, and this study selects the internationally known brand Shiatzy Chen, which is dedicated to the promotion of Chinese style design.

Selection of experimental products: Separately from the 15 brands mentioned above, official websites or shopping platforms were used as sales indicators. For the evaluation, we selected the top 5 sellers of the China-chic T-shirt products. The selection period was from 3 July 2021 to 1 January 2022. A total of 75 products were used as the pre-experimental products in this paper. Sales volume was used as an evaluation indicator, because a higher sales volume of a product, to a certain extent, indicates that the attractiveness of the product is more prominent, so it is preferred by many target consumers.

Determination of experimental products: The 75 pre-experimental products were organized and edited to produce 9 cm × 9 cm picture cards, and 5 China-chic T-shirt product designers were invited to score the attractiveness of the products. Finally, the 75 pre-experimental products were sorted according to the average value of the attractiveness characteristics. The top 31 products were selected as the official experimental products for the evaluation grid method interviews. The following chart shows a sample of 15 of these products.

#### 3.1.2. Participants of the study

The evaluation grid method was used to identify the attractiveness elements of the products and the psychological feelings associated with them by conducting in-depth interviews with people with high involvement in the product (Lu and Hsiao, 2019). In this paper, highly involved people with a certain understanding of China-chic T-shirt products were divided into two categories, designers and consumers, according to their backgrounds. In addition, Nielsen and Landauer (1993) found, through market research, that in the field of human–computer interactions, 76% of information available can be obtained by interviewing five skilled users of a product. To enhance the reliability of the interview results, a total of 12 interviewers were selected for this study, of which 5 designers (including three males and two females) and 7 consumers (including four males and three females) were invited. The selection principles of the interviewees were as follows: (1) The designers had to have at least 5 years of experience in the design of China-chic T-shirt products; (2) Consumers needed to have more than 3 years of experience in the purchase or use of China-chic T-shirt products; (3) The proportions of male and female interviewees were intended to be 50% each to reduce the interference of gender factors (Li et al., 2020).

#### 3.1.3. Experimental steps and the drawing of the evaluation structure diagram

In this study, we used the evaluation grid method to deconstruct the attractive elements of China-chic T-shirt products through interviews in a step-by-step manner. The experimental procedure was divided into three steps:

1. Step 1: One-on-one in-depth interviews were conducted with the participants using pre-prepared experimental sample cards of China-chic T-shirt products. Participants were asked to classify the 31 experimental samples into “like” and “dislike” categories according to their “preferences,” and the sample cards that they did not like were eliminated.
2. Step 2: In a pairwise comparison, participants were asked to explain the differences or reasons for their preferences for experimental products to establish the reasons for the participants’ original assessments in terms of the median item. Based on the original assessments, we further asked about and the abstract reasons (upper items) and concrete reasons (lower items) that contributed to the assessment of the items. A specific example is as follows: Researchers ask the participant, “What attracted you to Case Product 1 compared to Case Product 2”? Initially, participants answered, “I think the color matching of this T-shirt looks good,” and then researchers listed “product color matching” as the original factor contributing to the assessment of attractiveness (middle item). Based on the original assessment, researchers continued to ask, “What color match characteristics of the T-shirt product do you find attractive”? If the participant answered, “I like the red and green color match of this product,” then “red and green color match” was listed as a concrete reason for the product’s attractiveness (lower item). Finally, the researcher asked, “What do you find attractive about the red and green color scheme of the T-shirt product? Can you describe the feeling”? If respondents answered, “the color scheme of red and green gives me a sense of vitality,” then “sense of vitality” was listed as an abstract reason (upper item).
3. Step 3: Using the data obtained aforementioned, this paper continues to cross-compare the attractive elements proposed by participants through the method of focus group discussion, and merge all the elements with similar descriptions (for example, “Panda Pattern,” “Phoenix Pattern,” and “Koi Fish Pattern” are integrated into “Traditional Animal Pattern”), as well as add the number of occurrences after these elements, representing how many participants that have been mentioned the same elements. Subsequently, the present study places the upper items on the left, the middle items in the middle, and the lower items on the right, and then connects them with lines to illustrate the cause-and-effect relationships identified during the interviews. Consequently, a structural diagram of the evaluation structure is drawn.

**Figure 4:**
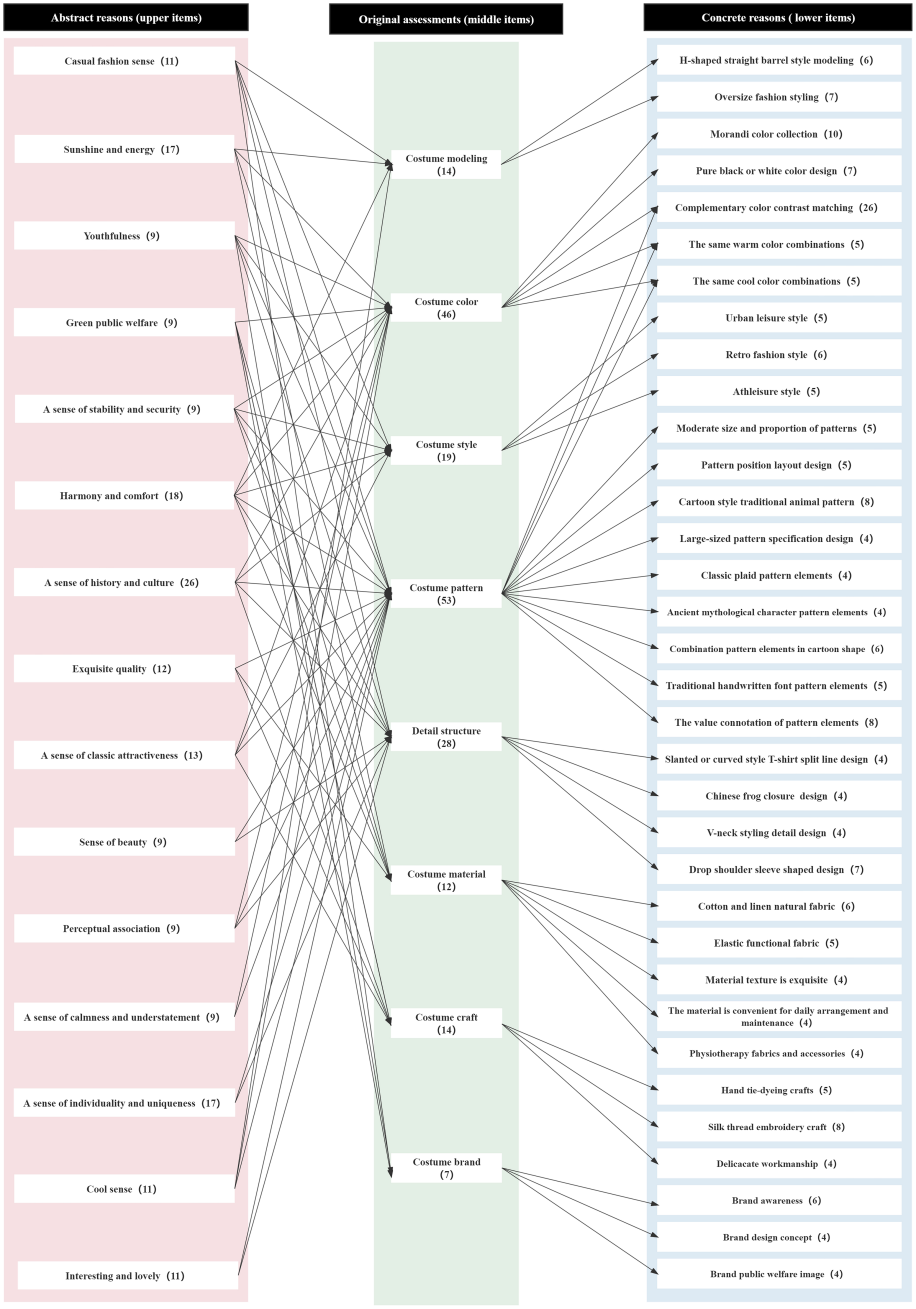
The overall evaluation structure diagram constructed for China-chic T-shirt products. Drawn by the author.

After carrying out the above steps, based on the interview data collected from 12 interviewees, we drew evaluation grid structural diagrams and then combined the 12 structural diagrams to build a preliminary overall evaluation grid structural diagram. Through convergence, 25 abstract reasons (upper items), 8 original assessments (middle items), and 48 concrete reasons (lower items) were summarized in the preliminary overall evaluation structure diagram. To get a clearer understanding of the attractiveness of China-chic T-shirt products, the Item average (Total frequency of items/number of items after summarization) was used as a filtering criterion, and the abstract reasons (upper items) and concrete reasons (lower items) were simplified and extracted (Shi, 2015). After calculating the average values, nine abstract reasons were used as screening conditions, and 15 abstract reasons were obtained after streamlining. Four concrete reasons were used as filtering conditions, and 34 concrete reasons were obtained after streamlining. After the attractiveness elements were extracted from each evaluation item by streamlining, the overall evaluation structure diagram of China-chic T-shirt products was drawn again, as shown in Figure 4 below and was used as the basis for subsequent research.

### 3.2. Analysis of the sample structure of the questionnaire

To further understand the structure of and connections between the attractiveness elements associated with China-chic T-shirt products and to extract the common factors among them, this phase of the study was based on the concrete reasons (lower items) summarized in the first phase of the evaluation grid method, and a reliability and validity analysis and exploratory factor analysis were conducted.

First, the 34 concrete reasons used as attractiveness elements were reordered randomly, and each concrete reason was converted into a question item in the questionnaire. Second, using a seven-point Likert scale, ranging from strongly disagree to strongly agree, the research respondents checked the boxes to represent to their own degrees of willingness. Finally, based on the questionnaire survey results, we integrated the reliability and validity analysis and factor analysis methods to extract the common factors in the attractiveness elements and name them.

In this study, we mainly collected data in the form of questionnaires. In total, 211 valid questionnaires were obtained. This meets the requirement for the number of samples suggested by scholars (Costello and Osborne, 2009). Of the 211 questionnaires, 111 were filled out by females and 100 by males. In terms of the age distribution, 87 respondents were younger than 25 years old, 45 respondents were 25–32 years old, 32 respondents were 33–39 years old, and 47 respondents were older than 39 years old. In terms of the education distribution, 46 people had a high school level of education or below, 63 people had a college level education, 66 people had undergraduate degrees, and 36 people had postgraduate degrees.

After the questionnaires had been collected, the first factor analysis revealed that the factor loadings of the two attractiveness elements “urban leisure style” and “retro fashion style” were not higher than 0.5, so they were eliminated. The data were then subjected to a second factor analysis, which yielded a KMO value of 0.846, higher than the test standard of 0.7 (Yong and Prarce, 2013). The Bartlett’s spherical test value was 3342.980, with a significance value of 0.000 (within the 0.05 significance level proposed by Kaiser), which was significant. The above results show that the data is suitable for factor analysis. Subsequently, we used the principal component analysis to conduct common factor extraction. The first extraction factor was the most representative, and the second factor was the second-most representative. Common factors with eigenvalues greater than one were retained, and those with eigenvalues of less than one were not considered. As a result, seven common factors were obtained, explaining 65.685% of the cumulative variance. After the principal component analysis had been used to extract the seven factors, the maximum variance method was used to continue the factor rotation. The rotated component matrix was obtained as a result (Table 1), making each factor’s representative meaning more prominent and easier to explain. Finally, the reliability and validity analysis revealed that the Cronbach’s alpha values of all seven factors were higher than 0.7, and the average variance extracted (AVE) values of each factor were greater than 0.5. Moreover, the square root values of the average variance extracted were greater than the correlations with other factors (as shown in Table 2), which verifies that the seven factors have good reliability while also indicating good discriminant validity among the factors (Bonett and Wright, 2015; Limon, 2020).

**Table 1:** Factor analysis.

Factors	Attractiveness Elements	Factor 1	Factor 2	Factor 3	Factor 4	Factor 5	Factor 6	Factor 7
Perceptual association features	Combination pattern elements in cartoon shape	0.827	0.147	0.109	0.194	0.095	0.056	0.015
	The value connotation of pattern elements	0.823	−0.003	0.134	0.048	0.019	−0.002	0.079
	Cartoon style traditional animal pattern	0.813	0.088	0.143	0.111	0.140	0.046	0.114
	Ancient mythological character pattern elements	0.804	0.015	0.077	0.094	0.015	0.017	0.153
	Traditional handwritten font pattern elements	0.770	−0.042	0.132	0.112	0.026	0.082	0.043
	Classic plaid pattern elements	0.735	0.090	0.212	0.083	0.166	0.066	−0.006
	Hand tie-dyeing crafts	0.681	0.060	0.078	0.096	0.042	−0.024	−0.005
	Chinese frog closure design	0.675	0.141	0.042	0.098	0.012	−0.022	0.059
Beauty features	Pattern position layout design	0.114	0.826	0.015	0.174	−0.038	0.075	0.054
	The same warm color combinations	0.063	0.811	0.105	0.034	0.069	0.104	0.114
	Pure black or white color design	0.049	0.800	0.083	0.040	0.067	−0.025	0.106
	Moderate size and proportion of patterns	0.120	0.743	0.122	0.032	0.038	0.100	0.168
	Morandi color collection	0.002	0.689	−0.003	0.127	0.024	0.231	0.090
	The same cool color combinations	0.069	0.655	0.131	−0.020	0.201	0.011	−0.129
Delicacy features	Drop shoulder sleeve shaped design	0.078	0.119	0.809	0.054	0.120	0.063	−0.004
	Material texture is exquisite	0.154	−0.011	0.804	0.013	−0.034	−0.009	0.068
	V-neck styling detail design	0.215	0.161	0.775	0.028	0.011	0.046	0.067
	Delicate workmanship	0.116	0.093	0.741	0.011	0.007	0.104	−0.021
	Silk thread embroidery craft	0.163	0.058	0.718	0.101	0.046	−0.006	0.084
Creativity features	Slanted or curved style T-shirt split line design	0.213	0.091	0.066	0.796	0.065	0.041	0.027
	Large-sized pattern specification design	0.080	0.067	0.043	0.784	0.099	0.050	0.136
	Complementary color contrast matching	0.186	0.117	0.054	0.747	0.035	0.138	−0.027
	Oversize fashion styling	0.249	0.058	0.042	0.720	0.322	0.079	0.066
Engineering features	Elastic functional fabric	0.158	0.153	0.113	0.143	0.793	0.121	0.169
	H-shaped straight barrel style modeling	0.022	0.073	0.034	0.178	0.759	0.113	−0.069
	The material is convenient for daily arrangement and maintenance	0.175	0.069	−0.015	0.072	0.756	0.195	0.112
Green features	Athleisure style	0.056	0.117	0.018	0.022	0.047	0.816	0.049
	Cotton and linen natural fabric	0.079	0.108	0.100	0.103	0.211	0.788	0.211
	Physiotherapy fabrics and accessories	−0.027	0.206	0.095	0.203	0.242	0.748	0.079
Brand image features	Brand Design Concept	0.042	0.083	0.055	0.044	0.054	0.078	0.839
	Brand public welfare image	0.137	0.071	0.076	0.021	−0.033	0.075	0.776
	Brand awareness	0.156	0.228	0.031	0.138	0.217	0.168	0.684

**Table 2:** Reliability and validity analysis.

	Cronbach’s Alpha	AVE	Perceptual association features	Beauty features	Delicacy features	Creativity features	Engineering features	Green features	Brand image features
Perceptual association features	0.915	0.590	0.768*						
Beauty features	0.868	0.573	0.205	0.757*					
Delicacy features	0.852	0.593	0.343	0.230	0.770*				
Creativity features	0.822	0.581	0.381	0.236	0.177	0.762*			
Engineering features	0.766	0.592	0.264	0.243	0.152	0.364	0.769*		
Green features	0.788	0.615	0.145	0.316	0.175	0.286	0.405	0.784*	
Brand image features	0.739	0.591	0.241	0.276	0.164	0.211	0.235	0.312	0.769*

The diagonal characters (marked with *) show the square root of the average variance extraction (AVE), and the lower triangle indicates the Pearson correlation coefficient.

Factor 1 is composed of 8 attractiveness elements: “Combination pattern elements in cartoon shape,” “The value connotation of pattern elements,” “Cartoon style traditional animal pattern,” “Ancient mythological character pattern elements,” “Traditional handwritten font pattern elements,” “Classic plaid pattern elements,” “Hand tie-dyeing crafts,” and “Chinese frog closure design.” For consumers, as these elements mainly emphasize their inner emotional attraction, they may give consumers a unique impression and feeling about the product through perceptual associations in the brain. Given this, we named this factor, which has the ability to influence people’s psycho-logical feelings, the perceptual association feature. For example, the cultural connotations expressed by the above-mentioned traditional pattern elements, modeling structures, or craft techniques will give people a nostalgic, storytelling, emotional feeling. In addition, the value connotation of the “alcohol allergy” text pattern will provide alcohol lovers with a health association and inspiration.

Factor 2 consists of 6 attractiveness elements: “Pattern position layout design,” “The same warm color combinations,” “Pure black or white color design,” “Moderate size and proportion of patterns,” “Morandi color collection,” and “The same cool color combinations.” This factor mainly emphasizes the use of moderate and ingenious pattern design (proportion, layout) and harmonious and unified color matching design in the product as a communication medium, so that consumers can form a visual perception of beauty and feel happy. Therefore, this factor was named the beauty feature.

Factor 3 is composed of five attractive elements: “Drop shoulder sleeve shaped design,” “Material texture is exquisite,” “V-neck styling detail design,” “Delicate workmanship,” and “Silk thread embroidery craft.” This factor focuses on a high level of craftsmanship, the use of delicate materials, and the detailed design of a product, giving the consumer an image of sophistication or refinement. This factor was named the delicacy features of the product.

Factor 4 is composed of 4 attractiveness elements: “Slanted or curved style T-shirt split line design,” “Large size pattern specification design,” “Complementary color contrast matching,” and “Oversize fashion styling.” This factor reflects the consumers’ pursuit of the creative attractiveness attribute of China-chic T-shirt products; that is, by strengthening the unique differentiated feeling of the product in design through factors such as the pattern (for example, the use of large size patterns to increase the eye-catching effect of the pattern), color (for example, the use of a color clashing design to enhance the sense of visual conflict), and shape (for example, the use of an oversize silhouette to highlight the trend and unisex style), creating a personality for the product and leaving an impression on consumers. This factor was named the creativity features of the product.

Factor 5 consists of 3 attractive elements: “Elastic functional fabric,” “H-shaped straight barrel style modeling,” and “The material is convenient for daily arrangement and maintenance.” This factor emphasizes the excellent engineering performance of a product, such as the elasticity of the material and the ease of daily arrangement and maintenance (for example, anti-wrinkle and anti-shrinkage) to satisfy the basic usage needs of consumers. This factor encompasses concepts such as the functionality and safety of the product, so it was named the engineering features of the product. The reason that “H-shaped straight barrel style modeling” was classified as an Engineering Feature is probably due to the fact that it is a semi-loose fitting silhouette that fits well, allowing ease of movement while also enabling quick ventilation and heat dissipation on hot summer days, which is a key concern for consumers.

Factor 6 is composed of 3 attractiveness elements: “Athleisure style,” “Cotton and linen natural fabric,” and “Physiotherapy fabrics and accessories.” This factor reflects that consumers influenced by the concept of green life hope to satisfy their pursuit of a green and healthy lifestyle through the products. Therefore, product style with sports and leisure or skin-friendly and comfortable attributes and certain physical therapy performance components in the product material are often consumers’ favorites. Given this, this factor was named the green features of the product.

Factor 7 is composed of three attractiveness elements: “brand design concept,” “Brand public welfare image,” and “brand awareness.” These elements belong to the brand image concept category and are commonly used to measure the brand image power. At the same time, this factor also reflects the important influence of brand image in consumers’ product purchasing decisions. Du and Du (2019) research pointed out that the brand image factors of common good (public welfare), empathy (idea recognition), and pleasant positive (higher popularity) bring happiness to consumers, thereby influencing their product purchasing behaviors. Therefore, this factor was named the brand image features of the product.

Through the above analysis, we obtained seven index dimensions, the “perceptual association features,” “beauty features,” “delicacy features,” “creativity features,” “engineering features“, “green features,” and “brand image features,” as well as 32 elements. We used these to establish the objective evaluation index for the attractiveness attribute of China-chic T-shirt products.

## 4. Case validation of the evaluation index of attractive attributes of China-chic T-shirt products

Through the above research and analysis, the evaluation indexes of the attractive attributes of China-chic T-shirt products were determined and constructed. Then, to evaluate the evaluation index’s validity and practical application value, we invited experts and scholars from the apparel field to select three representative China-chic T-shirt products that are currently on sale in the market as experimental samples. We also selected two consumer types with different decision-making personalities and thinking and feeling attributes to analyze the different perceptions of the constructed China-chic T-shirt product evaluation index.

### 4.1. Selection of survey objects and determination of case products

In order to test whether the evaluation index of the attractiveness attribute of China-chic T-shirt products can measure differences in product attractiveness traits, this study selected people with different decision-making personalities as the targets of comparison. The screening was conducted using the Myers–Briggs Type Indicator (MBTI) to differentiate between participants. The MBTI is a personality classification theoretical model that is now widely used in academic and business fields. The MBTI detects human personality differences that can be divided into four dimensions, as follows: (1) According to the direction in which spiritual energy is obtained, people are classified as extroverts and introverts; (2) According to the method of understanding the world and processing information, people are divided into perceptual and intuitive types; (3) Based on decision preferences, people divided into thinking and feeling types; and (4) According to their life attitudes, people are divided into judgment and perception types (Carlyn, 1977). The MBTI is used to measure people’s personality and behavioral preferences, and the results can help to explain why different people have different interests in different things or why different people are good at different jobs.

The overall evaluation of a product by a consumer is considered a decision-making behavior. Therefore, through a set of MBTI questionnaires with 28 questions, this study divided 25 interviewees into “thinking-oriented consumers” and “feeling-oriented consumers” according to their MBTI’s personality parameters in the decision-making mode. Among the 11 respondents who tended to have a thinking type of personality, five were male and six were female, and they had an age range of 20–45 years old; among the 14 respondents who tended to have a feeling type of personality, six were male and eight were female, and they had an age range of 19–42 years old.

In addition, to testing the differences in the participants’ perceptions, this study commissioned a research team composed of experts and scholars previously to select three representative China-chic T-shirt products sold in the market as tested products (Table 3). The selection criteria of the tested products include product brand awareness, brand image, product style, product sales volume and product design points, etc.

**Table 3:** Tested products.

Product Name: Blue Bird Embroidered Long Sleeve T-Shirt	Name: Bamboo Pattern Embroidery Knitted T-shirt	Product Name: Personality Stand Collar Off Shoulder T-shirt
Fabric composition: viscose fiber 66%, Linen 34%	Fabric composition: 100% cotton	Fabric composition: 100% cotton
Design features: Chinese tailoring, implicitness style, Embroidery of the bluebird pattern from the Classic of Mountains and Seas, Lace-up design, Chinese Frog Button, etc.	Design features: Bamboo pattern embroidery, Business casual, high quality, etc.	Design features: Personality and sexy, Immortal Crane Print, Chinese stand collar, Cut-out sleeve design, etc.
Brand information: Chinese original designer brand	Brand information: international luxury brand	Brand information: Chinese original designer brand

### 4.2. Case evaluation process and results

In recent years, a variety of quantitative methods have been applied to the evaluation of quantitative indicators in the decision-making field, including scoring method, ranking method, Delphi method, analytic hierarchy process, quality function deployment, data envelopment analysis, multi-criteria decision analysis, etc. Despite the fact that these traditional methods have a high practical value as their theoretical clarity and ability to analyze and explore complex decision-making problems using simple architectures, these quantitative methods do not take into account the subjectivity of human thinking and fuzzy characteristics that are uneasy to quantify. In particular, design decision-making is a multi-criteria decision-making management process, and the commonly used evaluation vocabulary in design is generally subjective, uncertain and ambiguous. Therefore, fuzzy analytic hierarchy process, as a quantitative method aimed at exploring the subjective or thinking processes of human beings and dealing with the ambiguity of human discourse meaning mathematically, can not only effectively contribute to the related deficiencies of traditional methods, but also used as the basis for the evaluation or decision-making of design issues (Chang, 1966).

This study used a fuzzy pairwise comparison scale based on the evaluation index of the attractive attributes of China-chic T-shirt products and evaluated the tested products through on-site interviews. In the process of filling out the scale, the researcher first introduced the attractive attribute evaluation index connotation and the case product and explained the content of the scale and how to fill out the scale to ensure that the respondents had a clear conceptual perception. Subsequently, the research team displayed the case products so that the interviewees could deeply understand the product details.

After all respondents had completed the questionnaire, fuzzy hierarchical analysis scoring matrices were created separately for the two decision-making personality groups according to the different products. Taking the Blue Bird Embroidered Long Sleeve T-Shirt as an example, the pairwise comparison rating matrix for the evaluation of attractiveness attributes of the 1st thinking type respondent (T1) was established, and the calculation process used was as follows.

(1) Construction of the fuzzy hierarchical analysis scoring matrix:
$$\begin{array}{c} \begin{array}{c} \begin{array}{cccccccc} & & & & \left[A\right] & & & \end{array}\\ \begin{array}{cccccccc} & D_{1} & D_{2} & D_{3} & D_{4} & D_{5} & D_{6} & D_{7} \end{array} \end{array}\\ \begin{array}{cc} \begin{array}{c} D_{1}\\ D_{2}\\ D_{3}\\ D_{4}\\ D_{5}\\ D_{6}\\ D_{7} \end{array} & \left|\begin{array}{ccccccc} 0.5 & 0.5 & 0.6 & 0.3 & 0.7 & 0.8 & 0.6\\ 0.5 & 0.5 & 0.5 & 0.6 & 0.5 & 0.6 & 0.5\\ 0.4 & 0.5 & 0.5 & 0.4 & 0.6 & 0.7 & 0.5\\ 0.7 & 0.4 & 0.6 & 0.5 & 0.7 & 0.7 & 0.6\\ 0.3 & 0.5 & 0.4 & 0.3 & 0.5 & 0.6 & 0.3\\ 0.2 & 0.4 & 0.3 & 0.3 & 0.4 & 0.5 & 0.3\\ 0.4 & 0.5 & 0.5 & 0.4 & 0.7 & 0.7 & 0.5 \end{array}\right| \end{array} \end{array}$$

In the matrix, *D*_1_ is the perceptual association feature; *D*_2_ is the beauty feature; *D*_3_ is the delicacy feature; *D*_4_ is the creativity feature; *D*_5_ is the engineering feature; *D*_6_ is the green feature; and *D*_7_ is the brand image feature.

(2) Matrix dimension: *n* = 7.
(3) Sum the matrix *A* by rows to obtain the matrix *R*, and transpose the matrix *R*.
$$R^{T}={\left(a_{i}\right)}_{7\times 1}=\left[4\;3.7\;3.6\;4.2\;2.9\;2.4\;3.7\right]$$
(4) Calculate the weight value of each index *W*_1_:
α=(n−1)/2=(7−1)/2=3
$$w_{i}=\frac{1}{n}-\frac{1}{2\alpha }+\frac{a_{i}}{n\alpha }$$
$$\begin{array}{l} W_{1}{}^{T}={\left(w_{i}\right)}_{7\times 1}\\ \;\;\;=\left[0.166\;7\;\;0.152\;4\;\;0.147\;6\;\;0.176\;2\;\;0.114\;3\;\;0.090\;5\;\;0.152\;4\right] \end{array}$$
(5) Construct the weight matrix *W*_2_:
$$w_{ij}=\alpha \left(w_{i}-w_{j}\right)+0.5$$
$$\begin{array}{cc} & \begin{array}{ccccccc} & & & \left[W_{2}\right] & & & \end{array}\\ & \begin{array}{ccccccc} \;\;\;D_{1\;\;\;\;\;} & \;D_{2\;\;\;\;\;\;} & \;D_{3\;\;\;\;\;} & \;D_{4\;\;\;\;\;} & \;D_{5\;\;\;\;} & \;D_{6\;\;\;\;} & \;D_{7\;} \end{array}\\ \begin{array}{c} D_{1}\\ D_{2}\\ D_{3}\\ D_{4}\\ D_{5}\\ D_{6}\\ D_{7} \end{array} & \left|\begin{array}{ccccccc} 0.500 & 0.543 & 0.557 & 0.471 & 0.657 & 0.729 & 0.543\\ 0.457 & 0.500 & 0.514 & 0.429 & 0.614 & 0.686 & 0.500\\ 0.443 & 0.486 & 0.500 & 0.414 & 0.600 & 0.671 & 0.486\\ 0.529 & 0.571 & 0.586 & 0.500 & 0.686 & 0.757 & 0.571\\ 0.343 & 0.386 & 0.400 & 0.314 & 0.500 & 0.571 & 0.386\\ 0.271 & 0.314 & 0.328 & 0.243 & 0.429 & 0.500 & 0.314\\ 0.457 & 0.500 & 0.514 & 0.429 & 0.614 & 0.686 & 0.508 \end{array}\right| \end{array}$$
(6) Calculate the consistency index CI:
$$CI\left(A,W_{2}\right)=\frac{\sum_{i=1}^{n}\sum_{j=1}^{n}\left|w_{ij}-a_{ij}\right|}{n^{2}}=0.0449$$

It can be seen from the calculation results that the consistency index is less than 0.1, which means that the questionnaire data reached an acceptable consistency standard, so the index weight value calculated above is valid.

We repeated the above steps, carried out weight calculations and consistency tests, and found the weight values of the evaluation indexes of the attractiveness attributes of the China-chic T-shirt products for 11 thinking-type respondents and 14 feeling-type respondents in the case of Blue Bird Embroidered Long Sleeve T-Shirt. Using the above steps, the index weights of each respondent were calculated for two cases, the bamboo pattern embroidery knitted T-shirt and the Personality stand collar off shoulder T-shirt, and the final calculation results are shown in Table 4.

**Table 4:** Contingency table of the evaluation index weights of T-shirt products with different design styles by respondents with different decision-making per-sonalities.

Number	Blue Bird Embroidered Long Sleeve T-Shirt							Bamboo Pattern Embroidery Knitted T-shirt							Personality Stand Collar Off Shoulder T-shirt						
	Perceptual association feature	Beauty feature	Delicacy feature	Creativity feature	Engineering feature	Green feature	Brand image feature	Perceptual association feature	Beauty feature	Delicacy feature	Creativity feature	Engineering feature	Green feature	Brand image feature	Perceptual association feature	Beauty feature	Delicacy feature	Creativity feature	Engineering feature	Green feature	Brand image feature
*T*_1_	0.1667	0.1524	0.1476	0.1762	0.1143	0.0905	0.1524	0.1048	0.1429	0.1571	0.1238	0.1762	0.1476	0.1476	0.1381	0.1333	0.1619	0.1381	0.1667	0.1143	0.1476
*T*_2_	0.1095	0.1476	0.1238	0.1905	0.1952	0.1333	0.1000	0.1000	0.1429	0.1333	0.1762	0.1714	0.1429	0.1333	0.1333	0.1524	0.1333	0.1476	0.1714	0.1381	0.1238
*T*_3_	0.1476	0.1429	0.1571	0.1333	0.1524	0.1286	0.1381	0.1286	0.1524	0.1333	0.1476	0.1476	0.1429	0.1476	0.1333	0.1238	0.1286	0.1714	0.1810	0.1476	0.1143
*T*_4_	0.0952	0.1476	0.1238	0.2095	0.1476	0.1667	0.1095	0.1048	0.1571	0.1286	0.1810	0.1381	0.1714	0.1190	0.0857	0.1333	0.1238	0.1810	0.2095	0.1619	0.1048
*T*_5_	0.1524	0.1476	0.1714	0.1286	0.1381	0.1143	0.1476	0.1286	0.1381	0.1333	0.1571	0.1476	0.1429	0.1524	0.1381	0.1143	0.1571	0.1667	0.1762	0.1333	0.1143
*T*_6_	0.1619	0.1810	0.1571	0.1810	0.1000	0.0952	0.1238	0.1190	0.1524	0.1333	0.1190	0.1667	0.1476	0.1619	0.1095	0.1476	0.1476	0.1571	0.1714	0.1286	0.1381
*T*_7_	0.0857	0.1524	0.1429	0.1714	0.1714	0.1571	0.1190	0.1095	0.1381	0.1333	0.1619	0.1667	0.1524	0.1381	0.1000	0.1381	0.1571	0.1618	0.1620	0.1429	0.1381
*T*_8_	0.1672	0.1714	0.1278	0.1320	0.1619	0.1099	0.1298	0.1476	0.1524	0.1238	0.1048	0.1857	0.1429	0.1429	0.1333	0.1762	0.1381	0.2000	0.0952	0.1095	0.1476
*T*_9_	0.1143	0.1571	0.1381	0.1524	0.1524	0.1524	0.1333	0.1381	0.1429	0.1429	0.1381	0.1476	0.1524	0.1381	0.1286	0.1429	0.1429	0.1571	0.1476	0.1429	0.1381
*T*_10_	0.1046	0.1655	0.1074	0.1494	0.1093	0.2224	0.1415	0.0713	0.1438	0.1791	0.0976	0.1753	0.1423	0.1906	0.1644	0.1581	0.1037	0.2335	0.1802	0.0656	0.0945
*T*_11_	0.1619	0.1571	0.1095	0.1857	0.1143	0.1095	0.1619	0.0952	0.1381	0.1619	0.1048	0.1952	0.1571	0.1476	0.1333	0.1524	0.1143	0.1571	0.1714	0.1190	0.1524
*^−^X_T_*	0.1334	0.1566	0.1370	0.1645	0.1415	0.1345	0.1324	0.1134	0.1456	0.1418	0.1374	0.1653	0.1493	0.1472	0.1271	0.1429	0.1371	0.1701	0.1666	0.1285	0.1285
*F*_1_	0.1476	0.1476	0.1381	0.1476	0.1333	0.1333	0.1524	0.1524	0.1381	0.1429	0.1429	0.1286	0.1381	0.1571	0.1476	0.1381	0.1381	0.1381	0.1333	0.1524	0.1524
*F*_2_	0.1810	0.1810	0.1238	0.1190	0.1381	0.0762	0.1810	0.0905	0.1381	0.1762	0.1333	0.1714	0.1238	0.1667	0.1524	0.1619	0.0952	0.1905	0.1095	0.1048	0.1857
*F*_3_	0.1905	0.1476	0.0905	0.1476	0.1429	0.0810	0.2000	0.1619	0.1571	0.1333	0.1238	0.1286	0.1667	0.1286	0.1095	0.1143	0.1333	0.1714	0.1571	0.1810	0.1333
*F*_4_	0.2048	0.1714	0.1286	0.1476	0.0905	0.0810	0.1762	0.0905	0.1667	0.1857	0.1095	0.1571	0.1905	0.1000	0.2000	0.1143	0.0952	0.1476	0.1476	0.0952	0.2000
*F*_5_	0.2238	0.1524	0.0952	0.1476	0.0952	0.0714	0.2143	0.1810	0.1571	0.1476	0.1429	0.0762	0.1143	0.1810	0.1810	0.1619	0.1048	0.1810	0.1238	0.0857	0.1619
*F*_6_	0.1000	0.1333	0.1667	0.1476	0.1762	0.1619	0.1143	0.1238	0.1286	0.1381	0.1381	0.1667	0.1524	0.1524	0.1238	0.1381	0.1476	0.1667	0.1429	0.1429	0.1381
*F*_7_	0.1524	0.1476	0.1476	0.1190	0.1381	0.1333	0.1619	0.1476	0.1381	0.1381	0.1143	0.1524	0.1429	0.1667	0.1429	0.1476	0.1333	0.1667	0.1238	0.1476	0.1381
*F*_8_	0.1857	0.1381	0.1333	0.0952	0.1571	0.1190	0.1714	0.1190	0.1286	0.1619	0.1095	0.1762	0.1857	0.1190	0.1476	0.1476	0.0952	0.1810	0.1143	0.1810	0.1333
*F*_9_	0.1762	0.1667	0.1143	0.1571	0.1286	0.1000	0.1571	0.1048	0.1381	0.1667	0.1095	0.1476	0.1619	0.1714	0.1333	0.1667	0.1095	0.1857	0.1143	0.1429	0.1476
*F*_10_	0.2286	0.1667	0.0952	0.1524	0.1048	0.0905	0.1619	0.2000	0.1381	0.1810	0.1095	0.1143	0.0810	0.1762	0.1905	0.1524	0.1000	0.1762	0.1095	0.1190	0.1524
*F*_11_	0.2238	0.1381	0.1048	0.1762	0.1143	0.1048	0.1381	0.2238	0.1524	0.1619	0.1190	0.1000	0.0905	0.1524	0.2000	0.1619	0.1143	0.1667	0.0857	0.1143	0.1571
*F*_12_	0.1571	0.1619	0.1429	0.1143	0.1524	0.1333	0.1381	0.1571	0.1143	0.1714	0.1143	0.1571	0.1429	0.1429	0.1619	0.1238	0.1095	0.1619	0.1381	0.1333	0.1714
*F*_13_	0.1905	0.1476	0.1524	0.0905	0.1190	0.1429	0.1571	0.1437	0.1986	0.1722	0.1008	0.1287	0.1553	0.1007	0.1905	0.1429	0.0857	0.2000	0.1381	0.1143	0.1286
*F*_14_	0.1762	0.1429	0.1190	0.1667	0.1524	0.0810	0.1619	0.1143	0.1286	0.1619	0.1238	0.1714	0.1619	0.1381	0.1429	0.1429	0.1571	0.1286	0.1286	0.1524	0.1476
*^−^X*_F_	0.1813	0.1531	0.1252	0.1377	0.1316	0.1078	0.1633	0.1436	0.1445	0.1599	0.1208	0.1412	0.1434	0.1467	0.1589	0.1439	0.1156	0.1687	0.1262	0.1333	0.1534
*M*	0.1592	0.1547	0.1306	0.1501	0.1362	0.1202	0.1490	0.1297	0.1450	0.1516	0.1285	0.1523	0.1461	0.1469	0.1442	0.1435	0.1256	0.1694	0.1448	0.1312	0.1419

*T* represents thinking-type respondents; *F* represents feeling-type respondents; ^−^*X_T_* is the weight value average for thinking-type respondents; ^−^*X_F_* is the weight value average for feeling-type respondents; *M* is the average weight value of all respondents.

## 5. Sensitivity analysis

In this study, to further understand whether respondents with different decision-making personalities had differences in the evaluation indexes of the attractive attributes of China-chic T-shirt products for different product cases, a two-way ANOVA with the mixed design was conducted with the results of the weight scores of the above seven index constructs, and the results obtained are shown in Table 5.

**Table 5:** Summary table of the two-factor ANOVA with a mixed design for the evaluation index of attractive attributes of China-chic T-shirt products.

Dimensions of the index	Source of variation	Type IIISS	Degrees of freedom	Mean of square	*F* value	Value of *p* (Significance)	*Post Hoc* analysis	Partial *η*^2^
Perceptual association feature	Decision-making personality	0.025	1	0.025	14.493	0.001*	*F* > *T*	0.387
	Design style	0.010	2	0.005	8.634	0.001*	*K*_1_ > *K*_3_ > *K*_2_	0.273
	Personality*Style	0.001	2	0.001	1.001	0.375		0.042
	Within	0.066	69					
	Between respondents	0.039	23	0.002				
	Residual	0.027	46	0.001				
Beauty feature	Decision-making personality	0.000	1	0.000	0.096	0.759		0.004
	Design style	0.002	2	0.001	4.248	0.020*	*K*_1_ > *K*_2_ > *K*_3_	0.156
	Personality*Style	0.000	2	0.000	0.140	0.870		0.006
	Within	0.017	69					
	Between respondents	0.007	23	0.000				
	Residual	0.010	46	0.000				
Delicacy feature	Decision-making personality	0.000	1	0.000	1.522	0.230		0.062
	Design style	0.008	2	0.004	9.211	0.000*		0.286
	Personality*Style	0.005	2	0.003	5.809	0.006*		0.202
	Within	0.028	69					
	Between respondents	0.007	23	0.000				
	Residual	0.021	46	0.000				
Creative feature	Decision-making personality	0.004	1	0.004	10.336	0.004 *	*T* > *F*	0.310
	Design style	0.020	2	0.010	15.240	0.000 *	*K*_3_ > *K*_1_ > *K*_2_	0.399
	Personality*Style	0.002	2	0.001	1.529	0.228		0.062
	Within	0.039	69					
	Between respondents	0.009	23	0.000				
	Residual	0.030	46	0.001				
Engineering feature	Decision-making personality	0.011	1	0.011	15.325	0.001 *	*T* > *F*	0.400
	Design style	0.003	2	0.002	2.968	0.061		0.114
	Personality*Style	0.003	2	0.001	2.474	0.095		0.097
	Within	0.044	69					
	Between respondents	0.017	23	0.001				
	Residual	0.027	46	0.001				
Green feature	Decision-making personality	0.001	1	0.001	1.340	0.259		0.055
	Design style	0.008	2	0.004	5.684	0.006*	*K*_2_ > *K*_3_ > *K*_1_	0.198
	Personality*Style	0.003	2	0.002	2.367	0.105		0.093
	Within	0.057	69					
	Between respondents	0.025	23	0.001				
	Residual	0.032	46	0.001				
Brand image feature	Decision-making personality	0.006	1	0.006	10.782	0.003*		0.319
	Design style	0.001	2	0.000	0.787	0.461		0.033
	Personality*Style	0.003	2	0.002	3.891	0.027*		0.145
	Within	0.033	69					
	Between respondents	0.013	23	0.001				
	Residual	0.020	46	0.000				

*indicates that the value of *p* is less than 0.05, with significant difference. *T* is the thinking-type respondent; *F* is the feeling-type respondent. *K*_1_ is the Blue Bird Embroidered Long Sleeve T-Shirt; *K*_2_ is the bamboo pattern embroidery knitted T-shirt; *K*_3_ is the personality stand collar off shoulder T-shirt.

The results of the perceptual association feature analysis show that the main effects of the different decision-making personality factors (*F*(1, 23) = 14.493, *p* = 0.01 < 0.05) and the different design style factors (*F*(2, 46) = 8.634, *p* = 0.01 < 0.05) were significantly different, while the interaction effect between the decision-making personality and design style was not significant. This indicates a significant difference in the degree of perception of the product’s perceptual associative features between thinking-type respondents and the feeling-type respondents, and the *post hoc* comparison results indicate that the feeling-type respondents have a higher degree of perception for the perceptual associative features than the thinking-type respondents. At the same time, significant differences in the perceived degree of the perceptual association traits of the respondents to the three different styles of products were also identified. The degree, from high to low, was represented by the Blue Bird Embroidered Long Sleeve T-Shirt, the Personality stand collar off shoulder T-shirt, and the bamboo pattern embroidery knitted T-shirt.

In the analysis of the beauty features, the main effects of the different design style factors (*F*(2, 46) = 4.248, *p* = 0.020 < 0.05) showed significant differences, but the different decision-making personality factor (*F*(1, 23) = 0.096, *p* = 0.759 > 0.05) and the interaction effect between the two (*F*(2, 46) = 0.140, *p* = 0.870 > 0.05) were not shown to have a significant relationship. The post-hoc comparison results showed that the perceived degree of beauty features for the case products, from the highest to the lowest, was the Blue Bird Embroidered Long Sleeve T-Shirt, the bamboo pattern embroidery knitted T-shirt, and then the personality stand collar off shoulder T-shirt.

The results of the creative feature analysis showed that the main effects of the different decision-making personality factors (*F*(1, 23) = 10.336, *p* = 0.004 < 0.05) and the different design style factors (*F*(2, 46) = 15.240, *p* = 0.000 < 0.05) showed significant differences. In contrast, the interaction effect between them was not significant. Among the decision-making personality aspects, the results of the post-hoc comparison show that the thinking-type respondents felt the creative features to a greater extent than the feeling-type respondents. In terms of the design style, the post-hoc comparison results show that the respondents had the strongest feelings about the creative features of the personality stand collar off shoulder T-shirt, followed by the Blue Bird Embroidered Long Sleeve T-Shirt, and the lowest feelings were associated with the bamboo pattern embroidery knitted T-shirt.

The analysis of the engineering features presented very different results from the beauty feature analysis. In terms of perceptions of these features, a significant difference was found for the main effect of different decision-making personality factors [*F*(1, 23) = 15.325, *p* = 0.001 < 0.05], but the main effect of different design style factors [*F*(2, 46) = 2.968, *p* = 0.061 > 0.05] and the effect of the interaction of the decision-making personality and design style [*F*(2, 46) = 2.474, *p* = 0.095 > 0.05] did not reach significance. This indicates that there was no difference in the respondents’ perception degrees regarding the engineering features of the three design case products. However, there was a difference in the perception level of the engineering features between respondents with different decision-making personalities, and post-hoc comparative analysis revealed that thinking-type respondents perceived the engineering features to a greater extent than the feeling-type respondents.

The analysis results for the green features were the same as for the beauty features. Among the three factors, only the main effect of the different design style factor [*F*(2, 46) = 5.684, *p* = 0.006 < 0.05] showed significant differences, while the main effect of the different decision-making personality factor [*F*(1, 23) = 1.340, *p* = 0.259 > 0.05] and the interaction effect for them [*F*(2, 46) = 2.367, *p* = 0.105 > 0.05] did not reach significance. The results of the post-hoc comparative analysis showed that the respondents’ degree of perception of the green features for the three case products, from highest to lowest, was the bamboo pattern embroidery knitted T-shirt, personality stand collar off shoulder T-shirt, and then the Blue Bird Embroidered Long Sleeve T-Shirt.

As for delicacy and brand image features, since the two-way ANOVA results for both factors showed significant differences in the interaction effects between different decision-making personalities and different design styles [*F*(2, 46) = 5.809, *p* = 0.006 < 0.05; *F*(2, 46) = 3.891, *p* = 0.027 < 0.05], it was necessary to conduct a simple main effect analysis to explore the interaction.

This study used the GLM (General Linear Model) grammar to conduct a simple main effect test on two index dimensions, the delicacy features and brand image features. The analysis results are shown in the Table 6. To control the expansion of the error rate of type I, when using the familywise error rate, five consecutive simple main effects were tested, so the significance level needed to be changed to 0.05/5 = 0.01. In the delicacy feature test, there was a significant difference in the perceived degree of the delicacy features for the three different styles of products among the feeling-type respondents [*F*(2, 46) = 20, *p* = 0.000 < 0.01]. After a *post hoc* comparative analysis, it was found that feeling-type respondents had the highest degree of delicacy feature perception for the bamboo pattern embroidery knitted T-shirt, followed by the Blue Bird Embroidered Long Sleeve T-Shirt and then the personality stand collar off shoulder T-shirt. Subsequently, a significant difference in the perceived degree for the delicacy features was identified between respondents with different decision-making personalities for the personality stand collar off shoulder T-shirt [*F*(1, 69) = 7.5, *p* = 0.008 < 0.01], where the thinking-type respondents felt it to a greater extent than the feeling-type respondents. In the simple main effect test of the brand image feature, the error problem was taken into account, so we also determined that the thinking-type respondents [*F*(2, 46) = 5, *p* = 0.011] showed a significant difference in the perception of the brand image features for the three different styles of products. After a post-hoc comparative analysis, it was found that the thinking-type respondents had the highest degree of perception for the brand image features for the bamboo pattern embroidery knitted T-shirt, followed by the Blue Bird Embroidered Long Sleeve T-Shirt and the lowest for the personality stand collar off shoulder T-shirt. Finally, in the test of the two design cases of the Blue Bird Embroidered Long Sleeve T-Shirt [*F*(1, 69) = 15, *p* = 0.000 < 0.01] and the personality stand collar off shoulder T-shirt [*F*(1, 69) = 15, *p* = 0.002 < 0.01], a significant difference in the degree of feeling of the brand image features was identified among different decision-making personalities respondents. In both cases, the degree of sense was higher among the feeling-type respondents than the thinking-type respondents.

**Table 6:** Summary table of the simple main effects of the delicacy features and brand image features.

Dimensions of the index	Source of variation	Type IIISS	Degrees of freedom	Mean of square	*F*-value	Value of *p* (Significance)	*Post Hoc* analysis
Delicacy features	Design style (dependent factor)						
	Thinking-type	0.000167	2	0.00008345	0.208	0.813	
	Feeling-type	0.015	2	0.008	20	0.000*	*K*_2_ > *K*_1_ > *K*_3_
	Error (residual)	0.021	46	0.0004			
	Decision-making personality (independent factor)						
	Blue Bird Embroidered Long Sleeve T-Shirt	0.001	1	0.001	2.5	0.118	
	bamboo pattern embroidery knitted T-shirt	0.002	1	0.002	5	0.029	
	personality stand collar off shoulder T-shirt	0.003	1	0.003	7.5	0.008*	*T* > *F*
	Error (residual)	0.028	69	0.0004			
Brand image features	Design style (dependent factor)						
	Thinking-type	0.003	2	0.002	5	0.011*	*K*_2_ > *K*_1_ > *K*_3_
	Feeling-type	0.002	2	0.001	2.5	0.093	
	Error (residual)	0.020	46	0.0004			
	Decision-making personality (independent factor)						
	Blue Bird Embroidered Long Sleeve T-Shirt	0.006	1	0.006	15	0.000*	*F* > *T*
	bamboo pattern embroidery knitted T-shirt	0.000001	1	0.0000017	0.004	0.950	
	personality stand collar off shoulder T-shirt	0.004	1	0.004	10	0.002*	*F* > *T*
	Error (residual)	0.033	69	0.0004			

*indicates that the *p*-value is less than 0.05, indicating a significant difference. *T* is the thinking-type respondents; *F* is the feeling-type respondents. *K*_1_ is the Blue Bird Embroidered Long Sleeve T-Shirt; *K*_2_ is the bamboo pattern embroidery knitted T-shirt; *K*_3_ is the personality stand collar off shoulder T-shirt.

By integrating the results of the ANOVA for the above seven factors, we obtained the following results.

Firstly, the three case products had differences in the attractive attribute evaluation index of China-chic T-shirt products, which proves that the attractive attribute evaluation index of China-chic T-shirt products does have a good ability to distinguish the attractive attributes of products with different design styles. For example, the Blue Bird Embroidered Long Sleeve T-Shirt had the highest weight on the perceptual association features (0.1592) and the second highest on the beauty features (0.1547), indicating that although the respondents were in agreement regarding the beauty features of this product, such as its good shape proportion and fine design details, they still focused on the cultural connotation and storytelling revealed by the product. The bamboo pattern embroidery knitted T-shirt showed the opposite results to the Blue Bird Embroidered Long Sleeve T-Shirt, with respondents paying more attention to the functional aspects of this T-shirt, such as the delicacy features (0.1516) and the engineering features (0.1523), than to the beauty and other features. Finally, among the attractive attributes of the personality stand collar off shoulder T-shirt, the highest weight was given to the creative features (0.1694), followed by the perceptual association features (0.1442). Therefore, for the three case products, except for the engineering feature, which did not differ significantly between products, all of the indexes showed significant differences from each other in terms of leading situations. The reason why only the engineering features were not significant is possibly because the three case products were carefully selected by industry experts and scholars. Therefore, the critical engineering performance of these three products can be guaranteed. There may have been some differences between respondents with different decision-making personalities for a specific product, but the differences were still not significant in terms of the overall feeling of the three products. If, among the products tested, there were products with relatively average or even poor functional characteristics, differences may have been seen.

Secondly, different decision-making personalities were also associated with differences in the evaluation index of the attractive attributes of China-chic T-shirt products, which verifies that the evaluation index of the attractive attributes of China-chic T-shirt products does have an ideal detection ability for distinguishing consumers with different decision-making personalities. For example, for the respondents with the thinking-type decision-making personality, the seven evaluation indexes were ranked according to the weight value, as follows: the engineering features (0.4734), the creative features (0.472), the beauty features (0.4451), the delicacy features (0.4081), the green features (0.4123), the brand image features (0.4081), and the perceptual association features (0.3739). This means that during the case product evaluation, the engineering feature of the product is often the most important and attractive point for the respondents with a thinking-type of decision-making personality. In contrast to respondents with a thinking-type of decision-making personality, respondents with a feeling-type of decision-making personality perceived the degree of the product indexes, in descending order of weight value, as follows: the perceptual association features (0.4838), the brand image features (0.4634), the beauty features (0.4415), the creativity features (0.4272), the delicacy features (0.4007), the engineering features (0.3990), and the green features (0.3845). This shows that associative attributes, such as cultural connotations, symbolism, and storytelling of products, are the most attractive to respondents with a feeling-type of decision-making personality. In conclusion, in the examination of different decision-making personalities, except for two index dimensions, the green features and the beauty features, which did not show significant differences, the index dimensions showed a mutual leading situation. The green feature and the beauty feature indexes did not show significant differences, similar to the above engineering feature. For example, from the comparison of the weight rankings of the two types of decision-making personality index dimensions, it was found that the beauty features ranked in third place, which indicates that the three case products all have high beauty characteristics and can all resonate with the respondents with different decision-making personalities. However, the difference is that the green features were not found to be significant, which may be related to the weakness of the three case products in terms of product green attributes. From the above comparison of the weighting ranks for the two types of decision-making personality index dimensions, it can be found that the green features ranked relatively low for both the respondents with thinking-type of decision-making personality and for the respondents with feeling-type of decision-making personality. Therefore, if among the products tested, there had been products with more obvious green characteristics, a difference may have been seen.

## 6. Conclusion and recommendations

In the era of emotion and esthetic economy, “attractiveness” has become a key factor in the innovative design of products. Products can evoke people’s feelings through shape and can indicate the characteristics of both the product and the consumer. Successful product design considers the users’ inner preferences and goes beyond practical value. A design trend of the 21st century is to incorporate “attractiveness” into product design and communicate with the users’ spirits. Starting from the sustainable development of the attractiveness consumption of China-chic T-shirt products, based on Miryoku engineering and the comprehensive use of various quantitative analysis techniques, this study aimed to build an evaluation index of the attractiveness attributes of China-chic T-shirt products to provide an important basis for enterprises and designers to improve product development and promote the sustainable development of product consumption. The conclusions of this study are summarized as follows:

1. This study explored the attractiveness elements of China-chic T-shirt products through the Miryoku engineering evaluation grid method and the exploratory factor analysis method and extracted the corresponding attractiveness factors according to the attributes of the elements. The study concludes that the attractive attributes of China-chic T-shirt products include 15 abstract reasons, eight original assessments, and 34 concrete reasons. Through extraction, these were classified as seven attractive factors and 32 attractive elements, including the perceptual association feature, beauty feature, delicacy feature, creativity feature, engineering feature, green feature, and brand image feature, and used to build an evaluation index system for the attractive attributes of China-chic T-shirt products.
2. This research used the fuzzy analytic hierarchy process to calculate the weight values. After the questionnaire interviews, fuzzy hierarchical analysis scoring matrixes were established, the weight of each factor was determined, and consistency tests were carried out. Then, the weight values of the evaluation indexes of the attractive attributes of the China-chic T-shirt products of the interviewed samples were calculated.
3. This study verified the practical value of the evaluation index of the attractive attributes of China-chic T-shirt products through actual case studies. The analysis of differences in perception was performed by applying a two-way ANOVA with a mixed design. The analysis results show that the evaluation index of the attractive attributes of the China-chic T-shirt products has a good ability to differentiate between the attractive attributes of different design style products and the respondents with different decision-making personalities.

The China-chic T-shirt product attractive attributes evaluation index will allow designers to design products according to potential customers’ preferences, avoiding misalignment between design and demand, product homogenization, and other phenomena. Similarly, related enterprises can use the China-chic T-shirt product attractive attribute evaluation index to classify the product. A design or product can be assessed to determine its unique product attractive characteristics according to the China-chic T-shirt product attractive attribute evaluation index in advance. This method can be used by market planning personnel to carry out market segmentation and competitor analysis as a reference. Finally, the design department of China-chic T-shirt enterprises can also use this index as a reference for advance planning and measurement of the whole design strategy.

At the same time, the study has the following research limitations and future directions: Firstly, this study was based on the use of the Miryoku engineering evaluation grid method to identify the attractive elements of China-chic T-shirt products. Future studies may combine the Kano two-dimensional quality model and its improvement model, the importance-performance analysis, and other research methods to explore optimization strategies to improve the attributes of China-chic T-shirt products from different resource-type enterprises. Secondly, since the target population of this study is limited to the Chinese population, it is suggested that other populations are included in the future to understand the preferences and needs for China-chic T-shirt products by other global populations, which will help relevant companies to implement brand internationalization strategies.

## Data availability statement

The raw data supporting the conclusions of this article will be made available by the authors, without undue reservation.

## Author contributions

HL: conceptualization, writing – original draft preparation, writing – review and editing. CG: resources and translation. BZ: supervision and funding acquisition. All authors have read and agreed to the published version of the manuscript.

## Funding

This study was supported by the funding of National Social Science Foundation of China (grant numbers 20BG119 and 20BG119).

## Conflict of interest

The authors declare that the research was conducted in the absence of any commercial or financial relationships that could be construed as a potential conflict of interest.

## Publisher’s note

All claims expressed in this article are solely those of the authors and do not necessarily represent those of their affiliated organizations, or those of the publisher, the editors and the reviewers. Any product that may be evaluated in this article, or claim that may be made by its manufacturer, is not guaranteed or endorsed by the publisher.
